# Metabolic Mediators of the Effects of Family History and Genetic Risk Score on Coronary Heart Disease—Findings From the Malmö Diet and Cancer Study

**DOI:** 10.1161/JAHA.116.005254

**Published:** 2017-03-20

**Authors:** Josef Fritz, Dov Shiffman, Olle Melander, Hayato Tada, Hanno Ulmer

**Affiliations:** ^1^ Department of Medical Statistics, Informatics and Health Economics Medical University of Innsbruck Austria; ^2^ Quest Diagnostics San Juan Capistrano CA; ^3^ Department of Clinical Sciences Lund University Malmö Sweden; ^4^ Department of Internal Medicine Skåne University Hospital Malmö Sweden; ^5^ Department of Cardiovascular and Internal Medicine Kanazawa University Graduate School of Medicine Kanazawa Japan

**Keywords:** coronary heart disease, epidemiology, family history, genetic association, risk factor, Epidemiology, Risk Factors, Genetics, Coronary Artery Disease

## Abstract

**Background:**

Family history of coronary heart disease (CHD) as well as genetic predisposition to CHD assessed by a genetic risk score (GRS) are predictors of CHD risk. It is, however, uncertain to what extent these risk predictors are mediated by major metabolic pathways.

**Methods and Results:**

Total effects of self‐reported family history and a 50‐variant GRS (GRS50), as well as effects mediated by apolipoprotein B and A‐I (apoB, apoA‐I), blood pressure, and diabetes mellitus, on incidence of CHD were estimated in 23 595 participants of the Malmö Diet and Cancer study (a prospective, population‐based study). During a median follow‐up of 14.4 years, 2213 participants experienced a first CHD event. Family history of CHD and GRS50 (highest versus other quintiles) were associated with incident CHD, with hazard ratios of 1.52 (95% CI: 1.39–1.65) and 1.53 (95% CI: 1.39–1.68), respectively, after adjusting for age, sex, and smoking status. Small proportions of the family history effect were mediated by metabolic risk factors: 8.3% (95% CI: 5.8–11.7%) by the apoB pathway, 1.7% (95% CI: 0.2–3.4%) by apoA‐I, 8.5% (95% CI: 5.9–12.0%) by blood pressure, and 1.5% (95% CI: −0.8% to 3.8%) by diabetes mellitus. Similarly, small proportions of GRS50 were mediated: 8.1% (95% CI: 5.5–11.8%) by apoB, 1.2% (95% CI: 0.5–3.0%) by apoA‐I, 4.2% (95% CI: 1.3–7.5%) by blood pressure, and −0.9% (95% CI: −3.7% to 1.6%) by diabetes mellitus.

**Conclusions:**

A fraction of the CHD risk associated with family history or with GRS50 is mediated through elevated blood lipids and hypertension, but not through diabetes mellitus. However, a major part (≥80%) of the genetic effect operates independently of established metabolic risk factor pathways.

## Introduction

Family history of coronary heart disease (CHD)^1^, ^2^, ^3^ as well as genetic predisposition to CHD directly assessed by genotyping and expressed as genetic risk score (GRS) for CHD^4^, ^5^, ^6^, ^7^ are strong predictors of CHD risk. Among others, Barrett‐Connor and Khaw already showed that family history predicts cardiovascular risk independently of established metabolic risk factors.^8^, ^9^, ^10^, ^11^, ^12^, ^13^ Similarly, it was later shown that GRSs are associated with CHD independently of established risk factors.^5^, ^14^, ^15^ However, a portion of the risk may well be explained or mediated through metabolic risk factor pathways. The fraction of risk associated with family history and GRS that is mediated through metabolic risk factor pathways has not been adequately reported in the medical literature.

Previous studies demonstrated that family history of CHD or GRS was significantly associated with CHD after adjusting for risk factors such as blood lipids, hypertension, and diabetes mellitus (DM).^5^, ^8^, ^9^, ^10^, ^11^, ^12^, ^13^, ^14^, ^15^ However, metabolic risk factors may not be confounders of genetic risk, but potential intermediate variables or mediators of the causal pathway between exposure (family history of CHD or GRS) and outcome (CHD incidence). A metabolically unhealthy person with a family history of CHD may suffer from hypertension or hypercholesterolemia; thus that person's predisposition to CHD is mediated through these risk factors. In contrast, a metabolically healthy person may have an increased genetic risk without elevated risk factor levels. The first case is an example of an indirect effect mediated by intermediate variables, while the second case is an example of a direct effect of the exposure on the outcome. The sum of the indirect and direct effects is the total effect. In contrast to adjustment for risk factors, statistical mediation analysis quantifies the magnitudes of total, direct, and indirect effects.

In the present study, we estimated the total effects of family history and of GRS, as well as the direct effect and indirect effects mediated by elevated blood lipids, hypertension, and DM on the incidence of CHD. Analyses were performed in the Malmö Diet and Cancer (MDC) study, a prospective, population‐based study providing the most up‐to‐date information on genetic CHD risk for 23 595 men and women. For this purpose, we applied recently developed methods^16^, ^17^ in the framework of causal inference.

## Methods

### Data Source and Study Population

The MDC study is a community‐based, prospective observational study of 30 447 participants drawn from about 230 000 residents of Malmö, Sweden. Men and women aged 45 to 73 years were invited to participate and were enrolled between 1991 and 1996. Details of the MDC study design have been previously reported.^18^, ^19^

Participants completed a baseline examination that included a blood draw and a questionnaire with assessment of cardiovascular risk factors, including cigarette smoking, family history of myocardial infarction, lipid‐lowering therapy, antihypertension therapy, and antidiabetic therapy.

Of note, apolipoprotein A‐I (apoA‐I) and apolipoprotein B (apoB) plasma levels were measured rather than high‐density lipoprotein (HDL) and low‐density lipoprotein (LDL) cholesterol levels. DM was self‐reported based on a physician's diagnosis of DM or use of antidiabetic medication. Self‐reported family history was based on the response of participants to a questionnaire about whether their mother, father, or sibling had a history of myocardial infarction.

Genotypes of the MDC study participants were determined using a multiplex method that combines polymerase chain reaction, allele‐specific oligonucleotide ligation assays, and hybridization to oligonucleotides coupled to Luminex^®^ 100TM xMAPTM microspheres (Luminex, Austin, TX).^20^ Subsequently, a genetic risk score accounting for 50 CHD‐related single nucleotide polymorphisms (SNPs) 50‐variant genetic risk score (GRS50) was calculated, as explained in more detail in the work of Tada et al.^21^ In short, each study participant received a score equal to the sum of the number of risk alleles for each SNP weighted by the log of the odds ratio reported with the SNP in the original report.

The primary end point of the study was time to first occurrence of CHD. Incident CHD was defined as coronary revascularization, fatal or nonfatal myocardial infarction, or death attributable to ischemic heart disease. Events were identified by comparison with 3 registers—the Swedish Hospital Discharge Register, the Swedish Cause of Death Register, and the Swedish Coronary Angiography and Angioplasty Registry (SCAAR)—via ICD‐9 codes 410, 412, and 414, ICD‐10 codes I21, I22, I23, and I24, procedure codes 3065, 3066, 3068, 3080, 3092, 3105, 3127, and 3158 (the Op6 system), and FN (the KKÅ97 system).^22^

After we excluded participants who were not genotyped, had prevalent CHD, or had missing information, 23 595 participants remained eligible for analyses in the current investigation. Details have been reported elsewhere.^21^

The MDC study was approved by the ethics committee at Lund University and performed in accordance with the 1964 Declaration of Helsinki and later amendments or comparable ethical standards. All participants provided written informed consent.

### Statistical Analysis

Study participants' characteristics and risk factor measurements were analyzed descriptively (mean and SD for continuous variables, and counts and percentages for categorical data). Associations of metabolic risk factors with incidence of CHD were evaluated in a multivariable Cox proportional hazards models adjusting for participants' age, sex, smoking, and use of antihypertensives.

Mediation analysis was performed using the natural effect model proposed by Lange et al,^16^, ^17^ based on the counterfactual framework. This approach offers a tool to decompose the total effect of a given exposure into a natural direct effect and a natural indirect effect through 1 or several mediators. The natural direct effect here is the effect one would expect if the sole difference between 2 individuals is the exposure (ie, family history of CHD or GRS50) and all risk factors are kept at the value they would naturally take. Detailed definitions of these effects have been described elsewhere.^23^, ^24^ Lange et al's method is designed for categorical exposures; therefore, we classified GRS50 into a low‐risk score category (quintile 1), an intermediate‐risk score category (quintiles 2–4), and a high‐risk score category (quintile 5). Mediation analysis was performed comparing the GRS50 risk categories high versus low and high versus intermediate and low combined. Figure shows the underlying model of our analyses depicted as a directed acyclic graph. We assessed the mediating role of apoA‐I, apoB, systolic blood pressure and hypertension treatment, and prevalent DM on the association between family history or GRS50 and CHD incidence, in the presence of the mediator‐outcome confounding variables age, sex, and smoking. For this purpose, systolic blood pressure and the information on hypertension treatment were aggregated into 1 combined variable: In a Cox model corrected for all confounders, the effect of hypertension treatment was estimated to be equivalent to an increase of 27.5 mm Hg in systolic blood pressure; therefore, for all participants using hypertension treatment, the systolic blood pressure value was increased by an extra 27.5 mm Hg and this modified value was used for all further mediation analyses. Similar models were calculated regarding family history as exposure and GRS50 as mediator and vice versa.

**Figure 1:**
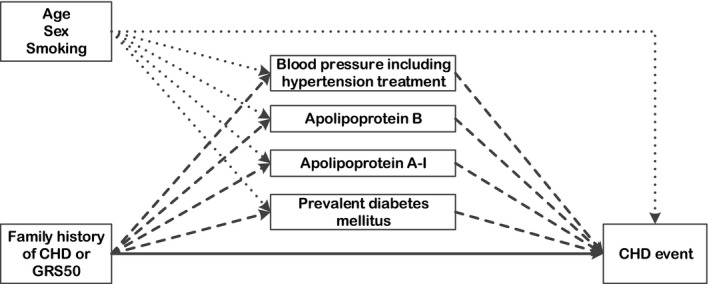
Causal diagram of the relations among genetic risk of CHD (exposure), metabolic risk factors of CHD (mediators), and CHD incidence (outcome) with measured confounders; metabolic risk factors are intermediate variables on the causal pathway between exposure (family history or GRS50) and outcome (CHD incidence). The direct effect is represented by the solid arrow and indirect effects are represented by dashed arrows; confounder pathways are depicted as dotted arrows. CHD indicates coronary heart disease; GRS50, 50‐variant genetic risk score.

Point estimates of the natural direct and natural indirect effects for the outcome CHD incidence were obtained by means of weighted regression models of the outcome on the exposure, the confounders, and additional counterfactual variables. Additional counterfactual variables taking the opposite (“counterfactual”) of the original exposure value were introduced in an extended set of the original data. Weights were derived from multivariable models regressing the mediators on the confounders and the exposure of interest (family history of CHD and GRS50, respectively). We used both Cox proportional hazards models quantifying relative risk in terms of hazard ratios (HRs), and additive hazards models with time‐independent effects^25^ quantifying absolute risk in terms of additional number of CHD events per 100 000 person‐years. In Data S1 we give annotated R code of our implementation of the statistical modeling procedure. The assumption of proportional hazards for the Cox models was visually checked by inspecting log(‐log) survival plots for all relevant variables. We also checked the assumption that each mediator is independent of the others, conditional on exposure and confounders in separate models.^17^

For HRs, the contribution of direct and indirect effects to the total effect of family history of CHD and GRS50 was calculated on the ln(HR) scale, since HRs are additive on this scale. Ninety‐five percent CIs were computed by bootstrapping using 2000 replications.

Possible effect modification by sex and age was examined by adding the respective multiplicative terms to the models. Because there was a significant interaction for indirect effects of apoA‐I (sex, *P*=0.025) and apoB (sex, *P*=0.026, and age, *P*<0.001), we repeated the previously described analyses separately for men and women, as well as for individuals <50 years of age.

It is worth noting that our approach is not equivalent to Mendelian randomization.^26^, ^27^ Neither self‐reported family history nor GRS qualify as instrumental variables, because of the direct effects operating independently of metabolic risk factors.^28^ A null or negative indirect effect in our setting does not mean that the variable concerned is not a risk factor for CHD.

Effects were considered statistically significant if null effects were not included in the 95% CI. All analyses were conducted in R, version 3.2.0.^29^

## Results

### Study Population

Demographic and risk factor characteristics of the 23 595 MDC study participants are shown in Table 1 (stratified by self‐reported family history and GRS50 categories). Mean age was 58.0 years, and 8973 (38.0%) of participants were male. By June 30, 2009, 2213 first CHD events were observed during a median follow‐up of 14.4 years. CHD incidence was markedly higher in participants with family history and those in the high GRS50 category. Participants with low GRS50 or without family history of CHD had lower levels of apoB and systolic blood pressure than those with high GRS50 or with family history of CHD. Those with family history of CHD were more likely to have prevalent DM than those without family history, although the differences between the groups were small.

**Table 1:** Baseline Characteristics According to Self‐Reported Family History of CHD and GRS50 Categories

	Family History		GRS50 Score		
	No (N=14 807)	Yes (N=8788)	Low (N=4719)	Intermediate (N=14 157)	High (N=4719)
Age, y	57.8 (7.7)	58.4 (7.7)	58.2 (7.8)	58.0 (7.7)	57.9 (7.7)
Men, n (%)	5963 (40.3)	3010 (34.3)	1829 (38.8)	5342 (37.7)	1802 (38.2)
Body mass index, kg/m^2^	25.7 (4.0)	25.8 (4.0)	25.7 (4.0)	25.7 (4.0)	25.8 (4.0)
Systolic blood pressure, mm Hg	140.5 (20.2)	142.1 (20.0)	140.8 (20.1)	141.0 (20.1)	141.8 (20.1)
Use of antihypertensives, n (%)	2307 (15.6)	1746 (19.9)	825 (17.5)	2417 (17.1)	811 (17.2)
Apolipoprotein A‐I, g/L	1.569 (0.281)	1.574 (0.279)	1.573 (0.279)	1.571 (0.283)	1.565 (0.275)
Apolipoprotein B, g/L	1.059 (0.259)	1.086 (0.262)	1.051 (0.260)	1.068 (0.261)	1.089 (0.259)
Prevalent diabetes mellitus, n (%)	566 (3.8)	364 (4.1)	178 (3.8)	575 (4.1)	177 (3.8)
Current smoker, n (%)	4268 (28.8)	2354 (26.8)	1316 (27.9)	3999 (28.2)	1307 (27.7)
Self‐reported family history of CHD, n (%)	0 (0.0)	8788 (100.0)	1552 (32.9)	5314 (37.5)	1922 (40.7)
GRS50	−0.045 (0.99)	0.075 (1.00)	−1.37 (0.42)	−0.02 (0.46)	1.43 (0.50)
Incident CHD event, n (%)	1215 (8.2)	998 (11.4)	318 (6.7)	1303 (9.2)	592 (12.5)
Median (mean) Follow‐up	13.87 (14.45)	13.65 (14.37)	14.47 (13.97)	14.41 (13.80)	14.39 (13.56)
Incidence rate from CHD per 100 000 person‐years	591.8	832.1	482.3	667.1	925.2

Data are presented as mean (SD) unless indicated. CHD indicates coronary heart disease; GRS50, 50‐variant genetic risk score.

All metabolic risk factors were associated with risk of incident CHD: the HRs per 1 SD increment were 1.28 (95% CI, 1.23–1.34) for systolic blood pressure, 1.32 (95% CI, 1.26–1.37) for ApoB, and 0.78 (95% CI, 0.74–0.82) for ApoA‐I; the HR for prevalent DM was 2.28 (95% CI, 1.99–2.61).

### Mediation Analysis: Total, Direct, and Indirect Effects of Family History of CHD

Family history of CHD was associated with incident CHD with an HR of 1.52 (95% CI, 1.39–1.65), after adjusting for age, sex, and smoking status. A fraction of this risk (20.0%, 95% CI, 14.8–26.4%) could be attributed to indirect pathways mediated by established metabolic risk factors. Specifically, 8.3% (95% CI, 5.8–11.7%) of the total effect was mediated through the apoB pathway, 1.7% (95% CI, 0.2–3.4%) through apoA‐I, and 8.5% (95% CI, 5.9–12.0%) through systolic blood pressure. The indirect effect through DM (1.5% [95% CI, −0.8% to 3.8%]) did not reach statistical significance. In absolute terms, family history of CHD was associated with 220 additional events of CHD per 100 000 person‐years at risk, of which 52 could be attributed to metabolic risk factor pathways; the most relevant risk factors apo‐B and systolic blood pressure added 20 and 24 additional events, respectively (Table 2).

**Table 2:** Total, Direct, and Indirect Effects of Family History on Incident CHD With Metabolic Mediators, Adjusted for Age at Baseline, Sex, and Smoking Status

Effects	Family History (Yes vs No)		
	Hazard Ratio (95% CI)	Proportion Explained (%) (95% CI)^a^	Additional Incident CHD Cases Per 100 000 Person‐Years at Risk^b^
Total effect	1.52 (1.39–1.65)	100.0%	269.7
Direct effect^c^	1.40 (1.28–1.52)	80.0% (73.6–85.2%)	220.2
Indirect effect, combined	1.09 (1.07–1.11)	20.0% (14.8–26.4%)	52.5
Indirect effect, through systolic blood pressure and hypertension treatment	1.04 (1.03–1.05)	8.5% (5.9–12.0%)	23.5
Indirect effect, through apoA‐I	1.01 (1.00–1.01)	1.7% (0.2–3.4%)	5.1
Indirect effect, through apoB	1.04 (1.03–1.05)	8.3% (5.8–11.7%)	19.8
Indirect effect, through diabetes mellitus	1.01 (1.00–1.02)	1.5% (−0.8% to 3.8%)	4.1

apoA‐I indicates apolipoprotein A‐I; apoB, apolipoprotein B; CHD, coronary heart disease.

a On ln(HR) scale.

b Estimates from additive hazards models with time‐independent effects.

c Effect of family history not mediated by the 4 analyzed risk factors.

When comparing men and women in stratified analyses, 20.8% (95% CI, 14.1–31.0%) of family history was mediated through metabolic risk factors in women versus 16.3% (95% CI, 8.3–27.6%) in men (Table S1). The effects of family history (total HR: 1.81 [95% CI, 1.31–2.53]; proportion mediated: 23.5% [95% CI, 10.5–51.6%]) were generally higher in individuals below 50 years of age as compared to the overall population (Table S2).

### Mediation Analysis: Total, Direct, and Indirect Effects of GRS50

GRS50 (highest versus other quintiles) was associated with incident CHD with an HR of 1.53 (95% CI, 1.39–1.68), after adjusting for age, sex, and smoking status. Only 12.6% (95% CI, 7.3–19.1%) of this risk could be attributed to indirect pathways mediated by established metabolic risk factors. Most of this attributable risk was mediated through the apoB pathway (8.1%, 95% CI, 5.5–11.8%), 1.2% (95% CI, −0.5% to 3.0%) through apoA‐I, and 4.2% (95% CI, 1.3–7.5%) through systolic blood pressure. No statistically significant mediation was observed for DM (−0.9% [95% CI, −3.7% to 1.6%]). In absolute terms, GRS50 was associated with 281 additional events of CHD per 100 000 person‐years at risk, of which 33 could be attributed to metabolic risk factor pathways; the most relevant risk factors apo‐B and systolic blood pressure added 20 and 12 additional events, respectively (Table 3).

**Table 3:** Total, Direct, and Indirect Effects of GRS50 on Incident CHD With Metabolic Mediators, Adjusted for Age at Baseline, Sex, and Smoking Status

Effects	GRS50 (High vs Low)			GRS50 (High vs Low/Intermediate)		
	Hazard Ratio (95% CI)	Proportion Explained (%) (95% CI)^a^	Additional Incident CHD Cases Per 100 000 Person‐Years at Risk^b^	Hazard Ratio (95% CI)	Proportion Explained (%) (95% CI)^a^	Additional Incident CHD Cases Per 100 000 Person‐Years at Risk^b^
Total effect	2.01 (1.76–2.30)	100.0%	469.3	1.53 (1.39–1.68)	100.0%	314.2
Direct effect^c^	1.87 (1.64–2.14)	89.3% (84.0–94.2%)	424.7	1.45 (1.32–1.59)	87.4% (80.9–92.7%)	281.2
Indirect effect, combined	1.08 (1.04–1.11)	10.7% (5.8–16.0%)	44.6	1.06 (1.03–1.08)	12.6% (7.3–19.1%)	33.0
Indirect effect, through systolic blood pressure and hypertension treatment	1.02 (1.01–1.04)	3.5% (1.0–5.9%)	16.2	1.02 (1.01–1.03)	4.2% (1.3–7.5%)	11.9
Indirect effect, through apoA‐I	1.01 (1.00–1.02)	1.1% (−0.2% to 2.6%)	5.3	1.01 (1.00–1.01)	1.2% (−0.5% to 3.0%)	3.6
Indirect effect, through apoB	1.04 (1.03–1.06)	6.0% (3.7–8.6%)	22.3	1.04 (1.02–1.05)	8.1% (5.5–11.8%)	19.9
Indirect effect, through diabetes mellitus	1.00 (0.99–1.02)	0.2% (−1.6% to 2.7%)	0.7	1.00 (0.98–1.01)	−0.9% (−3.7% to 1.6%)	−2.4

apoA‐I indicates apolipoprotein A‐I; apoB, apolipoprotein B; CHD, coronary heart disease; GRS50, 50‐variant genetic risk score.

a On ln(HR) scale.

b Estimates from additive hazards models with time‐independent effects.

c Effect of GRS50 not mediated by the 4 analyzed risk factors.

When comparing only the highest versus the lowest quintile, the total effect was more pronounced, with an HR of 2.01 (95% CI, 1.76–2.30). However, proportions mediated were similar (Table 3).

In sex‐specific analyses, 17.7% (95% CI, 9.0–30.9%) of GRS50 (highest versus other quintiles) was mediated through metabolic risk factors in women versus 7.4% (95% CI, −0.1% to 15.5%) in men (Table S3). The effects of GRS50 (total HR: 2.01 [95% CI, 1.40–2.78]) were similar regarding proportion mediated (10.1% [95% CI, −1.3% to 28.8%]) in individuals below 50 years of age as compared to the overall population (Table S4).

### Relationship of Family History of CHD With GRS50

An analysis of the relationship between family history of CHD and GRS50 showed that of the total HR associated with family history (1.52; 95% CI, 1.39–1.65) only 6.2% (95% CI, 4.3–8.7%) was mediated through GRS50 (Table S5), and of the total HR associated with GRS50 (1.53, 95% CI, 1.39–1.68; highest versus other quintiles), 4.2% (95% CI, 2.5–6.6%) was mediated by family history (Table S6).

## Discussion

The main purpose of the present work was to explore whether the previously identified associations between family history or GRS50 and CHD incidence are mediated through major metabolic risk factors. Specifically, we sought to decompose the total effect of family history and GRS50, respectively, on CHD risk into a direct part and distinct indirect parts via different risk factor pathways. Our findings demonstrate that some of the risk associated with family history as well as some of the risk conferred by GRS50 is mediated through known metabolic risk factors, specifically apolipoproteins and blood pressure, but we did not find evidence that DM mediates these effects. However, a major part (≥80%) of the family history effect and of the GRS50 effect operates independently of established metabolic risk factor pathways.

Recently, genetic risk scores were added to cardiovascular risk prediction, reflecting a partially different aspect of genetic information than reported family history.^6^, ^21^, ^30^ Through genome‐wide association studies, identification of CHD‐related SNPs has progressed rapidly. Consequently, CHD‐related SNPs have been aggregated in genetic risk scores, providing simple predictive measures of the risk of developing CHD.^4^, ^5^, ^6^, ^7^ The number of known CHD‐related SNPs used in GRSs has expanded from just 13 in 2010^4^ to 25 in 2011,^31^ and 46 in 2013.^5^ The 50‐SNP GRS used for this study was introduced in 2016 by Tada et al^21^ and represents the most comprehensive GRS at time of writing.

Attempts to relate CHD‐predicting SNPs to metabolic risk factors have been made in several studies.^5^, ^14^, ^15^, ^31^ The CARDIoGRAMplusC4D Consortium found in their study of 46 CHD‐predicting SNPs, a subset of the GRS50 used here, that 12 SNPs were associated with lipid traits, 5 with blood pressure, and none with DM, leaving the major part of SNPs operating independently of these 3 risk factors.^15^ Ganna et al came to similar conclusions that “most of the CHD loci are not involved in pathways perturbing currently known risk factors.”^5^ However, their methods did not allow quantification of the proportions mediated. Our findings, that about 9% of the total effect of the GRS50 was mediated by apolipoproteins, 4% by blood pressure, and none by DM, confirm their heuristic findings.

Other studies investigated the role of family history, either self‐reported or with validated parental event records, as a predictor for CHD.^1^, ^2^, ^3^, ^8^, ^9^, ^10^, ^11^, ^12^, ^13^ Sesso et al^1^ examined the association of self‐reported family history of myocardial infarction with risk of offspring cardiovascular disease using data from the Physicians' Health Study and the Women's Health Study. They adjusted for cardiovascular risk factors, but did not perform a mediation analysis. However, proportions mediated through cardiovascular risk factors can be estimated from their published results as a rough approximate measure. The results of Sesso et al^1^ were quantitatively similar to ours, specifically, the proportions mediated as converted were in a comparable range. Lloyd‐Jones et al^13^ examined the association between parental cardiovascular disease and CHD risk using data from the Framingham Heart Study where parental events were validated. In contrast to Sesso et al^1^ and our results, Lloyd‐Jones et al^13^ gave higher estimates of total effects, and the proportions mediated as estimated from these published results were higher as well. It is possible that the effects of family history and mediated proportions may be diluted because of the self‐reporting of family history. In line with our subgroup analysis of MDC study participants aged below 50 years (Table S2), participants' age may constitute another reason for differences in effect estimates, because family history more strongly predicts onset of CHD at a younger age.^21^, ^32^ Thus, the higher proportion mediated through risk factors in the Framingham Heart Study, which investigated a younger study population, seems plausible. Furthermore, the knowledge of an existing genetic risk may alter a person's health behavior and may thus lead to reduced risk factor levels^33^ and consequently to smaller proportions mediated. Finally, errors in the measurement of the mediators may also lead to an underestimation of the indirect effects.^34^

Mediation analysis applied on the relationship between family history and GRS50 suggests that these 2 measures of genetic risk are partly independent of each other. The proportions mediated by metabolic risk factors were stronger for family history than for GRS50 (20.0% versus 12.6%), mainly because blood pressure mediation was larger for family history than for GRS (8.5% versus 4.2%). The search for candidate genes for CHD is still ongoing;^30^ thus, the addition of new genetic variants to GRSs can alter the difference between the mediated fractions of family history and GRS50.

Our study has several strengths and some potential limitations. Major strengths are the prospective study design, the large sample size, and the length of follow‐up in the MDC study. In addition, we were able to use the newest available genetic data in CHD so far. The recently developed mediation analysis technique^16^, ^17^ allowed for the first time quantification of the mediated effects separately for each single metabolic factor. Limitations are that the study was conducted in Swedish middle‐aged individuals; hence, the generalizability to other ethnicities or other age groups is uncertain. Although genetic risk assessment could be useful in the young,^21^ this study population did not include individuals younger than 45 years and thus no risk estimates for this age group are possible. Overrepresentation of women in the MDC study cohort may influence the somewhat different effect estimates between men and women. A recently published study showed age‐ and sex‐related differences regarding metabolic mediation.^35^ LDL cholesterol and HDL cholesterol levels were not available for our study population; therefore, we used the available apoA‐I and apoB plasma levels as covariates in our established risk factors model. Although apoB is incorporated in a few lipoproteins in addition to LDL and apoA‐I does not completely represent HDL, it has been shown that apolipoproteins have equally strong predictive abilities for future CHD events as LDL cholesterol does.^36^, ^37^, ^38^

In conclusion, a fraction of the CHD risk associated with family history or with GRS50 is mediated through elevated blood lipids and hypertension, but not through DM. However, the major part of the genetic effect operates independently from the established metabolic risk factors, confirming the importance of the assessment of family history and genetic predisposition to CHD. Metabolically healthy individuals with genetic predisposition form an important group of individuals at risk for CHD, providing a major challenge for primary prevention. Therefore, intensified preventive measures in addition to risk factor surveillance and treatment may be of benefit in genetically predisposed individuals.

## Sources of Funding

The Malmö Diet and Cancer study was made possible by grants from the Swedish Cancer Society, the Swedish Medical Research Council, the Swedish Dairy Association, the Albert Påhlsson and Gunnar Nilsson Foundations, and the Malmö City Council. Melander is supported by the European Research Council (StG‐282255); the Swedish Heart and Lung Foundation, Swedish Research Council; the Novo Nordisk Foundation; the Skåne University Hospital donation funds; the Medical Faculty, Lund University; the Governmental funding of clinical research within the National Health Services; the Albert Påhlsson Research Foundation, Region Skåne; the King Gustav V and Queen Victoria Foundation; and the Marianne and Marcus Wallenberg Foundation. Melander is the recipient of an investigator‐initiated grant from Quest Diagnostics.

## Disclosures

Shiffman is an employee of Quest Diagnostics. The remaining authors have no disclosures to report.

## Supporting information


**Data S1.** Annotated R code of the statistical modelling procedure.
**Table S1.** Total, Direct, and Indirect Effects of Family History on Incident CHD With Metabolic Mediators, Adjusted for Age at Baseline and Smoking Status, Stratified by Sex
**Table S2.** Total, Direct, and Indirect Effects of Family History on Incident CHD With Metabolic Mediators, Adjusted for Age at Baseline, Sex, and Smoking Status, in a Subgroup of Individuals <50 Years of Age
**Table S3.** Total, Direct, and Indirect Effects of GRS50 (High Versus Low/Intermediate) on Incident CHD With Metabolic Mediators, Adjusted for Age at Baseline, and Smoking Status, Stratified by Sex
**Table S4.** Total, Direct, and Indirect Effects of GRS50 (High Versus Low/Intermediate) on Incident CHD With Metabolic Mediators, Adjusted for Age at Baseline, Sex, and Smoking Status, in a Subgroup of Individuals <50 Years of Age
**Table S5.** Total, Direct, and Indirect Effects of Family History on Incident CHD With Mediator GRS50, Adjusted for Age at Baseline, Sex, and Smoking Status
**Table S6.** Total, Direct, and Indirect Effects of GRS50 on Incident CHD With Mediator Family History, Adjusted for Age at Baseline, Sex, and Smoking Status
