# Supplementary material for: Metabolic Mediators of the Effects of Family History and Genetic Risk Score on Coronary Heart Disease—Findings From the Malmö Diet and Cancer Study
Source: J Am Heart Assoc. 2017 Mar 20;6(3):e005254. doi: 10.1161/JAHA.116.005254 (PMC5524031; doi:10.1161/JAHA.116.005254)
Supplement: Supplementary file 1 — Data S1. Annotated R code of the statistical modelling procedure. Table S1. Total, Direct, and Indirect Effects of Family History on Incident CHD With Metabolic Mediators, Adjusted for Age at Baseline and Smoking Status, Stratified by Sex Table S2. Total, Direct, and Indirect Effects of Family History on Incident CHD With Metabolic Mediators, Adjusted for Age at Baseline, Sex, and Smoking Status, in a Subgroup of Individuals <50 Years of Age Table S3. Total, Direct, and Indirect Effects of GRS50 (High Versus Low/Intermediate) on Incident CHD With Metabolic Mediators, Adjusted for Age at Baseline, and Smoking Status, Stratified by Sex Table S4. Total, Direct, and Indirect Effects of GRS50 (High Versus Low/Intermediate) on Incident CHD With Metabolic Mediators, Adjusted for Age at Baseline, Sex, and Smoking Status, in a Subgroup of Individuals <50 Years of Age Table S5. Total, Direct, and Indirect Effects of Family History on Incident CHD With Mediator GRS50, Adjusted for Age at Baseline, Sex, and Smoking Status Table S6. Total, Direct, and Indirect Effects of GRS50 on Incident CHD With Mediator Family History, Adjusted for Age at Baseline, Sex, and Smoking Status [file JAH3-6-e005254-s001.pdf]

Supplemental Material

Data S1.

In this appendix, we provide detailed annotated R code of how we implemented the natural effects model, the statistical procedure we used for our analysis, including the calculation of bootstrapping confidence intervals, exemplarily for the variable family history. This method was introduced by Lange et al. in 2014 (Lange T, Rasmussen M, Thygesen LC. Assessing natural direct and indirect effects through multiple pathways. Am J Epidemiol. 2014;179:513–8).

The data frame `myData` contains the following variables. Data originates from the MDCS cohort.

`ID`	Subject identifier
`age`	Age at baseline examination
`sex`	Sex (1=male, 2=female)
`smoking`	Indicator variable (0/1) of smoking status
`FH`	Reported family history of CHD (0=no, 1=yes)
`ApoA`	ApoA-I level at baseline examination
`ApoB`	ApoB level at baseline examination
`DM`	Prevalent diabetes mellitus at baseline examination (0=no, 1=yes)
`BP`	Systolic blood pressure at baseline examination
`time`	CHD event time or censor time (years after baseline examination)
`event`	event indicator (0= No CHD event, 1=CHD event)

Our aim is to quantify how much of the effect of family history of CHD is mediated through the apoA-I pathway, the apoB pathway, the DM pathway, and the BP pathway, in the presence of the mediator-outcome confounders age, sex, and smoking. More specifically, we want to obtain estimates of natural direct, natural indirect, and total effects conditional on the confounder variables. We refer to the work of Pearl for definitions of these effects (Pearl J. Causality: Models, reasoning and inference. Cambridge University Press. 2009. 2nd edn.).

In a first step, we regress each mediator on the exposure variable `FH`, while conditioning on the covariates `age`, `sex`, and `smoking`. For the continuous mediators, we choose linear regressions, for the dichotomous mediator `DM` a logistic regression. We also save the residual variances of the linear models since they will be needed for the computation of weights later on. Since being convenient for the weight calculation, also a new variable `FHtemp` is created.

```
myData$FHtemp <- myData$FH
fitApoA <- lm(ApoA ~ FHtemp+age+sex+smoking, data=myData)
fitApoAvariance <- summary(fitApoA)$sigma^2
fitApoB <- lm(ApoB ~ FHtemp+age+sex+smoking, data=myData)
fitApoBvariance <- summary(fitApoB)$sigma^2
fitBP <- lm(BP ~ FHtemp+age+sex+smoking, data=myData)
fitBPvariance <- summary(fitBP)$sigma^2
fitDM <- glm(DM ~ FHtemp+age+sex+smoking, data=myData,
family="binomial")
```

To assess if the mediators do indeed correspond to distinct non-intertwined causal pathways, these models can be extended to include also the respective other mediator variables. For the variable `ApoA`, for example, the respective model is

```
fitApoA_check <- lm(ApoA ~ FHtemp+age+sex+smoking+ApoB+DM+BP,
data=myData)
```

Small parameter estimates for the additional variables `ApoB`, `DM`, and `BP` as well as non-significant p-values, obtained for example by

```
anova(fitApoA_check)
```

are an indication of non-intertwined causal pathways. Analogous analyses were done for the remaining three mediators.

Next, we construct a new, expanded dataset, denoted `myDataExpanded`, with new auxiliary exposure variables `FHInd1`, `FHInd2`, `FHInd3`, and `FHInd4`, each corresponding to the value of the exposure relative to the respective indirect path. We replicate each observation

16 times and assign `FHInd1`, `FHInd2`, `FHInd3`, and `FHInd4` the values 0 or 1 in all possible combinations of these (2^4=16 combinations). This can be achieved by the following code:

```
levelsOfFH <- unique(myData$FH)
ds1 <- myData
ds2 <- myData
ds1$FHInd1 <- levelsOfFH[1]
ds2$FHInd1 <- levelsOfFH[2]
tempds <- rbind(ds1,ds2)
ds1 <- tempds
ds2 <- tempds
ds1$FHInd2 <- levelsOfFH[1]
ds2$FHInd2 <- levelsOfFH[2]
tempds <- rbind(ds1,ds2)
ds1 <- tempds
ds2 <- tempds
ds1$FHInd3 <- levelsOfFH[1]
ds2$FHInd3 <- levelsOfFH[2]
tempds <- rbind(ds1,ds2)
ds1 <- tempds
ds2 <- tempds
ds1$FHInd4 <- levelsOfFH[1]
ds2$FHInd4 <- levelsOfFH[2]
myDataExpanded <- rbind(ds1,ds2)
```

Now, it comes to the computation of the weights. The weights consist of a part corresponding to the ApoA-I level, a part corresponding to the ApoB level, a part corresponding to the DM status, and a part corresponding to systolic blood pressure. These parts are calculated consecutively via the following code (using the `predict` function) and are then combined.

The weight part corresponding to the apoA-I level:

```
myDataExpanded$FHtemp <- myDataExpanded$FH
tempDir1 <-
dnorm(myDataExpanded$ApoA,mean=predict(fitApoA,type="response",newda
ta=myDataExpanded),sd=sqrt(fitApoAvariance))
myDataExpanded$FHtemp <- myDataExpanded$FHInd1
tempIndir1 <-
dnorm(myDataExpanded$ApoA,mean=predict(fitApoA,type="response",newda
ta=myDataExpanded),sd=sqrt(fitApoAvariance))
myDataExpanded$weight1 <- tempIndir1/tempDir1
```

The weight part corresponding to the apoB level:

```
myDataExpanded$FHtemp <- myDataExpanded$FH
tempDir2 <-
dnorm(myDataExpanded$ApoB,mean=predict(fitApoB,type="response",newda
ta=myDataExpanded),sd=sqrt(fitApoBvariance))
myDataExpanded$FHtemp <- myDataExpanded$FHInd2
tempIndir2 <-
dnorm(myDataExpanded$ApoB,mean=predict(fitApoB,type="response",newda
ta=myDataExpanded),sd=sqrt(fitApoBvariance))
myDataExpanded$weight2 <- tempIndir2/tempDir2
```

The weight part corresponding to the DM status:

```
myDataExpanded$FHtemp <- myDataExpanded$FH
temp <- predict(fitDM,type="response",newdata=myDataExpanded)
tempDir3 <- ifelse(myDataExpanded$DM==1,temp,1-temp)
myDataExpanded$FHtemp <- myDataExpanded$FHInd3
temp <- predict(fitDM,type="response",newdata= myDataExpanded)
tempIndir3 <- ifelse(myDataExpanded$DM==1,temp,1-temp)
myDataExpanded$weight3 <- tempIndir3/tempDir3
```

The weight part corresponding to blood pressure:

```
myDataExpanded$FHtemp <- myDataExpanded$FH
tempDir4 <-
dnorm(myDataExpanded$BP,mean=predict(fitBP,type="response",newdata=
myDataExpanded),sd=sqrt(fitBPvariance))
myDataExpanded$FHtemp <- myDataExpanded$FHInd4
tempIndir4 <-
dnorm(myDataExpanded$BP,mean=predict(fitBP,type="response",newdata=m
yDataExpanded),sd=sqrt(fitBPvariance))
myDataExpanded$weight4 <- tempIndir4/tempDir4
```

The final weight is obtained by multiplying over the four parts:

```
myDataExpanded$weight <-
myDataExpanded$weight1*myDataExpanded$weight2*myDataExpanded$weight3
*myDataExpanded$weight4
```

The stability of the weights can be evaluated by drawing a histogram:

```
hist(myDataExpanded$weight)
```

Finally, natural direct and natural indirect effects can be computed using a natural effects model, for example a Cox proportional hazards model (function `coxph` of the R package `survival`) or an additive hazards model (function `aalen` of the R package `timereg`), applied on the weighted dataset just created.

```
library(survival)
coxph(Surv(time,event) ~
FH+FHInd1+FHInd2+FHInd3+FHInd4+cluster(ID)+age+sex+smoking,
data=myDataExpanded,weights=myDataExpanded$weight)
library(timereg)
aalen(Surv(time,event) ~
const(FH)+const(FHInd1)+const(FHInd2)+const(FHInd3)+const(FHInd4)+co
nst(age)+const(sex)+const(smoking),data=myDataExpanded,
clusters=myDataExpanded$ID, weights=myDataExpanded$weight, robust=T)
```

The parameter estimates for `FH` of these models yield estimates of the natural direct effect, and the parameter estimates for `FHInd1`, `FHInd2`, `FHInd3`, and `FHInd4` yield estimates of the natural indirect effects via apoA-I, apoB, DM, and blood pressure pathways (conditional on confounder variables age, sex, and smoking status).

Instead of relying on the robust standard errors as output by the functions `coxph`/`aalen`, we use bootstrapping to obtain 95% bootstrap percentile confidence intervals. This is done by encapsulating the previous code in a function, and then applying the function `boot` of the R package `boot`.

The following R code encapsulates the previous commands in the function `NaturalEffectsModel` for a Cox proportional hazards model as the natural effects model.

```
NaturalEffectsModel <- function(ds0,i) {
      library(survival)
      ds <- ds0[i,]
      #Mediator models
      ds$FHtemp <- ds$FH
      fitApoA <- lm(ApoA ~ FHtemp+age+sex+smoking, data=ds)
      fitApoAvariance <- summary(fitApoA)$sigma^2
      fitApoB <- lm(ApoB ~ FHtemp+age+sex+smoking, data=ds)
      fitApoBvariance <- summary(fitApoB)$sigma^2
      fitBP <- lm(BP ~ FHtemp+age+sex+smoking, data=ds)
      fitBPvariance <- summary(fitBP)$sigma^2
      fitDM <- glm(DM ~ FHtemp+age+sex+smoking, data=ds,
      family="binomial")
      #Expansion of dataset
      levelsOfFH <- unique(ds$FH)
      ds1 <- ds
      ds2 <- ds
      ds1$FHInd1 <- levelsOfFH[1]
```

```
ds2$FHInd1 <- levelsOfFH[2]

tempds <- rbind(ds1,ds2)

ds1 <- tempds

ds2 <- tempds

ds1$FHInd2 <- levelsOfFH[1]

ds2$FHInd2 <- levelsOfFH[2]

tempds <- rbind(ds1,ds2)

ds1 <- tempds

ds2 <- tempds

ds1$FHInd3 <- levelsOfFH[1]

ds2$FHInd3 <- levelsOfFH[2]

tempds <- rbind(ds1,ds2)

ds1 <- tempds

ds2 <- tempds

ds1$FHInd4 <- levelsOfFH[1]

ds2$FHInd4 <- levelsOfFH[2]

myDataExpanded <- rbind(ds1,ds2)

#Weight corresponding to apoA-I

myDataExpanded$FHtemp <- myDataExpanded$FH

tempDir1 <-

dnorm(myDataExpanded$ApoA,mean=predict(fitApoA,type="response",

newdata=myDataExpanded),sd=sqrt(fitApoAvariance))

myDataExpanded$FHtemp <- myDataExpanded$FHInd1

tempIndir1 <-

dnorm(myDataExpanded$ApoA,mean=predict(fitApoA,type="response",

newdata=myDataExpanded),sd=sqrt(fitApoAvariance))

myDataExpanded$weight1 <- tempIndir1/tempDir1

#Weight corresponding to apoB

myDataExpanded$FHtemp <- myDataExpanded$FH
```

```
tempDir2 <-
dnorm(myDataExpanded$ApoB,mean=predict(fitApoB,type="response",
newdata=myDataExpanded),sd=sqrt(fitApoBvariance))
myDataExpanded$FHtemp <- myDataExpanded$FHInd2
tempIndir2 <-
dnorm(myDataExpanded$ApoB,mean=predict(fitApoB,type="response",
newdata=myDataExpanded),sd=sqrt(fitApoBvariance))
myDataExpanded$weight2 <- tempIndir2/tempDir2
#Weight corresponding to DM status
myDataExpanded$FHtemp <- myDataExpanded$FH
temp <- predict(fitDM,type="response",newdata=myDataExpanded)
tempDir3 <- ifelse(myDataExpanded$DM==1,temp,1-temp)
myDataExpanded$FHtemp <- myDataExpanded$FHInd3
temp <- predict(fitDM,type="response",newdata= myDataExpanded)
tempIndir3 <- ifelse(myDataExpanded$DM==1,temp,1-temp)
myDataExpanded$weight3 <- tempIndir3/tempDir3
#Weight corresponding to blood pressure
myDataExpanded$FHtemp <- myDataExpanded$FH
tempDir4 <-
dnorm(myDataExpanded$BP,mean=predict(fitBP,type="response",newd
ata= myDataExpanded),sd=sqrt(fitBPvariance))
myDataExpanded$FHtemp <- myDataExpanded$FHInd4
tempIndir4 <-
dnorm(myDataExpanded$BP,mean=predict(fitBP,type="response",newd
ata= myDataExpanded),sd=sqrt(fitBPvariance))
myDataExpanded$weight4 <- tempIndir4/tempDir4
#Final weight
```

```
    myDataExpanded$weight <-
    myDataExpanded$weight1*myDataExpanded$weight2*myDataExpanded$we
    ight3*myDataExpanded$weight4
    #Final natural effects model
    fitNatEffMod <- coxph(Surv(time,event) ~
    FH+FHInd1+FHInd2+FHInd3+FHInd4+cluster(ID)+age+sex+smoking,
    data=myDataExpanded,weights=myDataExpanded$weight)
    #Calculation of natural direct, natural indirect, and total
    effects as well as percentages
    coef <- fitNatEffMod$coefficients
    HRTE <- exp(coef[1]+coef[2]+coef[3]+coef[4]+coef[5])
    HRNDE <- exp(coef[1])
    HRNIE <- exp(coef[2]+coef[3]+coef[4]+coef[5])
    HRNIE1 <- exp(coef[2])
    HRNIE2 <- exp(coef[3])
    HRNIE3 <- exp(coef[4])
    HRNIE4 <- exp(coef[5])
    HRTEpct <- log(HRTE)/log(HRTE)*100
    HRNDEpct <- log(HRNDE)/log(HRTE)*100
    HRNIEpct <- log(HRNIE)/log(HRTE)*100
    HRNIE1pct <- log(HRNIE1)/log(HRTE)*100
    HRNIE2pct <- log(HRNIE2)/log(HRTE)*100
    HRNIE3pct <- log(HRNIE3)/log(HRTE)*100
    HRNIE4pct <- log(HRNIE4)/log(HRTE)*100
    return(c(HRTE,HRNDE,HRNIE,HRNIE1,HRNIE2,HRNIE3,HRNIE4,HRNDEpct,
    HRNIEpct,HRNIE1pct,HRNIE2pct,HRNIE3pct,HRNIE4pct))
}
```

Bootstrap percentile confidence intervals can then be obtained by the function `bootstrap` as defined as follows:

```
bootstrap <- function(pathname,numit){
      results <- boot(myData,NaturalEffectsModel,R=numit,stype="i")
      TE <-boot.ci(results, type = c("perc"),index=1)
      NDE<-boot.ci(results, type = c("perc"),index=2)
      NIE<-boot.ci(results, type = c("perc"),index=3)
      NIE1<-boot.ci(results, type = c("perc"),index=4)
      NIE2<-boot.ci(results, type = c("perc"),index=5)
      NIE3<-boot.ci(results, type = c("perc"),index=6)
      NIE4<-boot.ci(results, type = c("perc"),index=7)
      NDEpct<-boot.ci(results, type = c("perc"),index=8)
      NIEpct<-boot.ci(results, type = c("perc"),index=9)
      NIE1pct<-boot.ci(results, type = c("perc"),index=10)
      NIE2pct<-boot.ci(results, type = c("perc"),index=11)
      NIE3pct<-boot.ci(results, type = c("perc"),index=12)
      NIE4pct<-boot.ci(results, type = c("perc"),index=13)
      sink(pathname,append=FALSE,split=FALSE)
      cat("\n\nNumber of bootstrapping iterations: ",numit)
      cat("\n\nTotal effect: ", round(TE$t0,2),"
      [",round(TE$percent[4],2),"-",round(TE$percent[5],2),"],
      ",round(100,2)," [",round(100,2),"-",round(100,2),"]\n",sep="")
        cat("Direct effect: ", round(NDE$t0,2),"
      [",round(NDE$percent[4],2),"-",round(NDE$percent[5],2),"],
      ",round(NDEpct$t0,2)," [",round(NDEpct$percent[4],2),"-
      ",round(NDEpct$percent[5],2),"]\n",sep="")
        cat("Indirect effect: ", round(NIE$t0,2),"
      [",round(NIE$percent[4],2),"-",round(NIE$percent[5],2),"],
```

```
",round(NIEpct$t0,2)," [",round(NIEpct$percent[4],2),"-
",round(NIEpct$percent[5],2),"]\n",sep="")
  cat("Indirect effect (Mediator 1): ", round(NIE1$t0,2),"
[",round(NIE1$percent[4],2),"-",round(NIE1$percent[5],2),"],
",round(NIE1pct$t0,2)," [",round(NIE1pct$percent[4],2),"-
",round(NIE1pct$percent[5],2),"]\n",sep="")
  cat("Indirect effect (Mediator 2): ", round(NIE2$t0,2),"
[",round(NIE2$percent[4],2),"-",round(NIE2$percent[5],2),"],
",round(NIE2pct$t0,2)," [",round(NIE2pct$percent[4],2),"-
",round(NIE2pct$percent[5],2),"]\n",sep="")
  cat("Indirect effect (Mediator 3): ", round(NIE3$t0,2),"
[",round(NIE3$percent[4],2),"-",round(NIE3$percent[5],2),"],
",round(NIE3pct$t0,2)," [",round(NIE3pct$percent[4],2),"-
",round(NIE3pct$percent[5],2),"]\n",sep="")
  cat("Indirect effect (Mediator 4): ", round(NIE4$t0,2),"
[",round(NIE4$percent[4],2),"-",round(NIE4$percent[5],2),"],
",round(NIE4pct$t0,2)," [",round(NIE4pct$percent[4],2),"-
",round(NIE4pct$percent[5],2),"]\n",sep="")
sink()
return()
}
```

The function `bootstrap` performs the bootstrapping with the number of iterations specified by the parameter `numit`, and writes the results of the natural effects model for family history of CHD in a text file in the folder `pathname`.

For our data, we get the following output which is also the basis of Table 2 of the main text:

```
Number of bootstrapping iterations:  2000
Total effect: 1.52 [1.39-1.65], 100 [100-100]
Direct effect: 1.4 [1.28-1.52], 79.96 [73.58-85.21]
```

```
Indirect effect: 1.09 [1.07-1.11], 20.04 [14.79-26.42]

Indirect effect (Mediator 1): 1.01 [1-1.01], 1.7 [0.2-3.39]

Indirect effect (Mediator 2): 1.04 [1.03-1.05], 8.32 [5.84-11.7]

Indirect effect (Mediator 3): 1.01 [1-1.02], 1.49 [-0.78-3.76]

Indirect effect (Mediator 4): 1.04 [1.03-1.05], 8.53 [5.94-11.93]
```

Table S1. Total, direct, and indirect effects of family history on incident CHD with metabolic mediators, adjusted for age at baseline and smoking status, stratified by sex

Effects	Men (N=8,973)		Women (N=14,622)	
	**Hazard Ratio (95% CI)**	**Proportion explained (%) (95% CI)†**	**Hazard Ratio (95% CI)**	**Proportion explained (%) (95% CI)†**
**Total effect**	1.42 (1.27 to 1.59)	100.0%	1.67 (1.45 to 1.92)	100.0%
**Direct effect***	1.34 (1.20 to 1.49)	83.7% (72.4% to 91.7%)	1.50 (1.30 to 1.72)	79.2% (69.0% to 85.9%)
**Indirect effect, combined**	1.06 (1.03 to 1.09)	16.3% (8.3% to 27.6%)	1.11 (1.08 to 1.15)	20.8% (14.1% to 31.0%)
**Indirect effect, through systolic blood pressure and hypertension treatment**	1.02 (1.01 to 1.04)	6.4% (2.2% to 12.0%)	1.05 (1.03 to 1.06)	8.8% (5.6% to 13.8%)
**Indirect effect, through apoA-I**	1.01 (1.00 to 1.02)	2.2% (-0.3% to 5.3%)	1.01 (1.00 to 1.02)	1.7% (-0.1% to 3.9%)
**Indirect effect, through apoB**	1.03 (1.01 to 1.04)	7.5% (3.8% to 12.8%)	1.04 (1.03 to 1.06)	7.7% (4.8% to 12.4%)
**Indirect effect, through diabetes mellitus**	1.00 (0.99 to 1.01)	0.2% (-3.5% to3.6%)	1.01 (1.00 to 1.03)	2.6% (-0.6% to 6.1%)

CI – confidence interval; CHD – coronary heart disease
*Effect of family history not mediated by the four analyzed risk factors.
†On ln(HR) scale.

Table S2. Total, direct, and indirect effects of family history on incident CHD with metabolic mediators, adjusted for age at baseline, sex, and smoking status, in a subgroup of individuals <50 years of age

Effects	<50 years of age (N=4,684)	
	Hazard Ratio (95% CI)	Proportion explained (%) (95% CI)†
**Total effect**	1.81 (1.31 to 2.53)	100.0%
**Direct effect***	1.58 (1.15 to 2.19)	76.5% (48.4% to 89.5%)
**Indirect effect, combined**	1.15 (1.07 to 1.24)	23.5% (10.5% to 51.6%)
**Indirect effect, through systolic blood pressure and hypertension treatment**	1.05 (1.02 to 1.09)	7.9% (2.8% to 19.9%)
**Indirect effect, through apoA-I**	1.02 (1.00 to 1.04)	3.0 (0.4% to 9.5%)
**Indirect effect, through apoB**	1.08 (1.03 to 1.13)	12.4% (4.7% to 30.0%)
**Indirect effect, through diabetes mellitus**	1.00 (0.97 to 1.03)	0.2% (-6.1% to 5.4%)

CI – confidence interval; CHD – coronary heart disease

*Effect of family history not mediated by the four analyzed risk factors.

†On ln(HR) scale.

Table S3. Total, direct, and indirect effects of GRS50 (high vs. low/intermediate) on incident CHD with metabolic mediators, adjusted for age at baseline, and smoking status, stratified by sex

Effects	Men (N=8,973)		Women (N=14,622)	
	**Hazard Ratio (95% CI)**	**Proportion explained (%) (95% CI)†**	**Hazard Ratio (95% CI)**	**Proportion explained (%) (95% CI)†**
**Total effect**	1.52 (1.35 to 1.70)	100.0%	1.56 (1.32 to 1.83)	100.0%
**Direct effect***	1.47 (1.31 to 1.65)	92.6% (84.53% to 100.1%)	1.44 (1.23 to 1.68)	82.3% (69.1% to 91.0%)
**Indirect effect, combined**	1.03 (1.00 to 1.06)	7.4% (-0.1% to 15.5%)	1.08 (1.04 to 1.13)	17.7% (9.0% to 30.9%)
**Indirect effect, through systolic blood pressure and hypertension treatment**	1.02 (1.00 to 1.04)	4.9% (0.5% to 10.0%)	1.02 (1.00 to 1.04)	3.7% (-0.1% to 8.3%)
**Indirect effect, through apoA-I**	1.00 (0.99 to 1.01)	-0.9% (-3.8% to 1.7%)	1.01 (1.00 to 1.02)	2.9% (0.3% to 6.2%)
**Indirect effect, through apoB**	1.02 (1.01 to 1.04)	5.8% (2.5% to 10.3%)	1.05 (1.03 to 1.07)	10.0% (5.5% to 17.0%)
**Indirect effect, through diabetes mellitus**	0.99 (0.98 to 1.00)	-2.5% (-6.6% to 0.8%)	1.00 (0.99 to 1.03)	1.1% (-3.6% to 6.0%)

CI – confidence interval; CHD – coronary heart disease
*Effect of GRS50 not mediated by the four analyzed risk factors.
†On ln(HR) scale.

Table S4. Total, direct, and indirect effects of GRS50 (high vs. low/intermediate) on incident CHD with metabolic mediators, adjusted for age at baseline, sex, and smoking status, in a subgroup of individuals <50 years of age

Effects	<50 years of age (N=4,684)	
	**Hazard Ratio (95% CI)**	**Proportion explained (%) (95% CI)†**
**Total effect**	2.01 (1.40 to 2.78)	100.0%
**Direct effect***	1.87 (1.30 to 2.59)	89.9% (71.2% to 101.3%)
**Indirect effect, combined**	1.07 (0.99 to 1.17)	10.1% (-1.3% to 28.8%)
**Indirect effect, through systolic blood pressure and hypertension treatment**	1.00 (0.97 to 1.03)	-0.2% (-6.4% to 5.0%)
**Indirect effect, through apoA-I**	1.00 (0.97 to 1.02)	-0.4% (-4.4% to 3.1%)
**Indirect effect, through apoB**	1.07 (1.02 to 1.14)	10.3% (3.1% to 24.9%)
**Indirect effect, through diabetes mellitus**	1.00 (0.97 to 1.04)	0.4% (-5.4% to 7.2%)

CI – confidence interval; CHD – coronary heart disease
*Effect of GRS50 not mediated by the four analyzed risk factors.
†On ln(HR) scale.

Table S5. Total, direct, and indirect effects of family history on incident CHD with mediator GRS50, adjusted for age at baseline, sex, and smoking status

Effects	Family history (yes vs. no)		
	**Hazard Ratio (95% CI)**	**Proportion explained (%) (95% CI)†**	**Additional incident CHD cases per 100,000 person-years at risk‡**
**Total effect**	1.52 (1.40 to 1.65)	100.0%	273.2
**Direct effect***	1.48 (1.37 to 1.61)	93.8% (91.4% to 95.7%)	255.7
**Indirect effect through GRS50**	1.03 (1.02 to 1.03)	6.2% (4.3% to 8.7%)	17.6

CI – confidence interval; CHD – coronary heart disease
*Effect of family history not mediated by GRS50.
†On ln(HR) scale.
‡Estimates from additive hazards models with time-independent effects.

Table S6. Total, direct, and indirect effects of GRS50 on incident CHD with mediator family history, adjusted for age at baseline, sex, and smoking status

	GRS50 (high vs. low)			GRS50 (high vs. low/intermediate)		
**Effects**	**Hazard Ratio (95% CI)**	**Proportion explained (%) (95% CI)†**	**Additional incident CHD cases per 100,000 person-years at risk‡**	**Hazard Ratio (95% CI)**	**Proportion explained (%) (95% CI)†**	**Additional incident CHD cases per 100,000 person-years at risk‡**
**Total effect**	2.00 (1.75 to 2.29)	100.0%	470.1	1.53 (1.39 to 1.68)	100.0%	315.6
**Direct effect***	1.94 (1.69 to 2.21)	95.0% (92.2% to 96.9%)	447.1	1.50 (1.36 to 1.65)	95.8% (93.4% to 97.4%)	304.0
**Indirect effect through family history**	1.04 (1.02 to 1.05)	5.0% (3.1% to 7.8%)	23.0	1.02 (1.01 to 1.03)	4.2% (2.5% to 6.6%)	11.6

CI – confidence interval; CHD – coronary heart disease
*Effect of GRS50 not mediated by family history.
†On ln(HR) scale.
‡Estimates from additive hazards models with time-independent effects.
